# Repeatability of two semi-automatic artificial intelligence approaches for tumor segmentation in PET

**DOI:** 10.1186/s13550-020-00744-9

**Published:** 2021-01-06

**Authors:** Elisabeth Pfaehler, Liesbet Mesotten, Gem Kramer, Michiel Thomeer, Karolien Vanhove, Johan de Jong, Peter Adriaensens, Otto S. Hoekstra, Ronald Boellaard

**Affiliations:** 1 Department of Nuclear Medicine and Molecular Imaging, Medical Imaging Center, University of Groningen, University Medical Center Groningen, Groningen, The Netherlands; 2 Faculty of Medicine and Life Sciences, Hasselt University, Agoralaan Building D, 3590 Diepenbeek, Belgium; 3 Department of Nuclear Medicine, Ziekenhuis Oost Limburg, Schiepse Bos 6, 3600 Genk, Belgium; 4 Department of Radiology and Nuclear Medicine, VU University Medical Center, Amsterdam, The Netherlands; 5 Department of Respiratory Medicine, Ziekenhuis Oost Limburg, Schiepse Bos 6, 3600 Genk, Belgium; 6 Department of Respiratory Medicine, AZ Vesalius Hospital, Hazelereik 51, 3700 Tongeren, Belgium; 7 Institute for Materials Research (IMO) - Division Chemistry, Hasselt University, Agoralaan Building D, 3590 Diepenbeek, Belgium

**Keywords:** Repeatability, Textural segmentation, Convolutional neural network, Tumor segmentation PET

## Abstract

**Background:**

Positron emission tomography (PET) is routinely used for cancer staging and treatment follow-up. Metabolic active tumor volume (MATV) as well as total MATV (TMATV—including primary tumor, lymph nodes and metastasis) and/or total lesion glycolysis derived from PET images have been identified as prognostic factor or for the evaluation of treatment efficacy in cancer patients. To this end, a segmentation approach with high precision and repeatability is important. However, the implementation of a repeatable and accurate segmentation algorithm remains an ongoing challenge.

**Methods:**

In this study, we compare two semi-automatic artificial intelligence (AI)-based segmentation methods with conventional semi-automatic segmentation approaches in terms of repeatability. One segmentation approach is based on a textural feature (TF) segmentation approach designed for accurate and repeatable segmentation of primary tumors and metastasis. Moreover, a convolutional neural network (CNN) is trained. The algorithms are trained, validated and tested using a lung cancer PET dataset. The segmentation accuracy of both segmentation approaches is compared using the Jaccard coefficient (JC). Additionally, the approaches are externally tested on a fully independent test–retest dataset. The repeatability of the methods is compared with those of two majority vote (MV2, MV3) approaches, 41%SUV_MAX_, and a SUV > 4 segmentation (SUV4). Repeatability is assessed with test–retest coefficients (TRT%) and intraclass correlation coefficient (ICC). An ICC > 0.9 was regarded as representing excellent repeatability.

**Results:**

The accuracy of the segmentations with the reference segmentation was good (JC median TF: 0.7, CNN: 0.73). Both segmentation approaches outperformed most other conventional segmentation methods in terms of test–retest coefficient (TRT% mean: TF: 13.0%, CNN: 13.9%, MV2: 14.1%, MV3: 28.1%, 41%SUV_MAX_: 28.1%, SUV4: 18.1%) and ICC (TF: 0.98, MV2: 0.97, CNN: 0.99, MV3: 0.73, SUV4: 0.81, and 41%SUV_MAX_: 0.68).

**Conclusion:**

The semi-automatic AI-based segmentation approaches used in this study provided better repeatability than conventional segmentation approaches. Moreover, both algorithms lead to accurate segmentations for both primary tumors as well as metastasis and are therefore good candidates for PET tumor segmentation.

## Introduction

Positron emission tomography in combination with computed tomography (PET/CT) using the tracer fluorodeoxyglucose (FDG) is an important imaging modality for cancer diagnosis, tumor staging, prognosis or treatment follow-up [1, 2]. The volume of the segmented tumor in the PET image, also known as metabolic active tumor volume (MATV) as well as the total MATV (TMATV—including metastasis and lymph nodes), is one important metric for the evaluation of therapy response [3]. Observed differences in MATV/TMATV should reflect actual tumor volume differences and not segmentation errors. Therefore, a repeatable segmentation is of utmost importance. Hereby, a repeatable segmentation refers to a segmentation algorithm leading to comparable results when applied on two consecutive PET/CT images of the same patient under the same physiological conditions. The implementation of a repeatable segmentation algorithm is not trivial due to the challenges associated with PET images. Among them are factors regarding the image quality, e.g., the low signal-to-noise ratio, low spatial resolution and partial volume effects. Especially for smaller lesions, the partial volume effect can reduce the apparent tumor uptake, making the lesion difficult to detect and segment.

Up to now, a manual segmentation by an expert or (if available) a consensus segmentation of several experts are considered as gold standard. However, manual segmentations have several drawbacks, e.g., they are time-consuming, non-reproducible and come with a high inter-observer variability [4–6]. A recent study also demonstrated that even the consensus of several observers results in a low repeatability compared to automated segmentations [7].

To overcome the limitations of manual segmentations and to increase repeatability, a large number of (semi-) automatic segmentation methods have been developed. The most basic and frequently used ones are thresholding algorithms defining voxels with an intensity value above a certain threshold as part of the tumor [8]. Also adaptive and iterative algorithms are available which adapt the threshold according to the actual image characteristics [9]. However, the performance of all these thresholding approaches depends on the scanner type, reconstruction algorithm, as well as image noise and have therefore limitations [10].

Therefore, more robust segmentation algorithms have been developed aiming to improve segmentation accuracy and repeatability. These include methods using the statistical properties of the image as well as learning-based methods [11, 12]. Nevertheless, most of these approaches have only been tested on limited datasets and are not widely available. Therefore, (semi-) automated segmentation methods used in the clinic are still mainly simple threshold-based approaches.

Due to the mentioned limitations of available segmentation algorithms, there is the need for new, more robust segmentation approaches. Artificial intelligence (AI)-based segmentations such as convolutional neural networks (CNN) have shown very promising results for various segmentation tasks [13] and yield great promise for the segmentation of tumors in PET images. However, only a few studies use AI-based segmentation approaches for metabolic active tumor segmentation in PET images. Moreover, most studies combine the information of PET and CT images in order to get reliable segmentation results [14] or use some post-processing for an improvement of CNN segmentations [15]. Algorithms classifying each voxel as tumor or non-tumor using textural features of voxel neighborhoods have been used for the segmentation of e.g., lung carcinoma or head-and-neck cancer [16–18]. All of these studies combine the information of PET and CT images. In many cases, the PET/CT is performed with a low-dose CT, the latter does not have an optimal image quality for segmentation purposes. Therefore, it is of interest to develop AI-based PET segmentations that rely on PET information only. Additionally, in previous papers, segmentation approaches were applied on primary tumors only, while for the calculation of TMATV, an accurate and repeatable segmentation of metastasis and lymph nodes is important. This task is especially challenging due to the small size of metastasis, different tumor-to-background ratios and different locations of the metastasis in the body.

While several studies already reported the segmentation accuracy of AI-based segmentation algorithm, to the best of our knowledge, no study reported yet the repeatability of those algorithms. In this study, we investigate the repeatability of two AI approaches especially built to segment both primary tumors and metastasis accurately and repeatably. We focus on the segmentation task and do not consider lesion detection. This study includes a textural feature-based segmentation approach as well as a 3D CNN. All algorithms are trained, validated and tested on a dataset of Non-Small-Cell-Lung-Cancer (NSCLC) patients. Moreover, the algorithms are applied to a fully independent test–retest dataset of ten NSCLC patients scanned on two consecutive days. The repeatabilities of the AI segmentation approaches are compared with those of conventional segmentation algorithms used in the clinic.

## Materials and methods

### Datasets

**Fig. 1:**
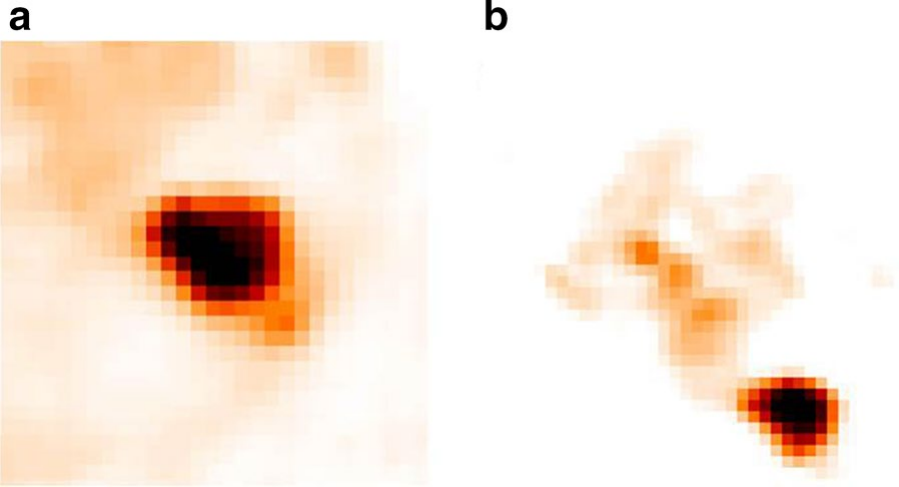
Two examples of a bounding box: A large bounding box is drawn around each lesion so that it also includes a large amount of background. For each lesion, the lesion is placed in a different position in the bounding box such that the CNN is not learning mainly the position of the lesion in the bounding box. On the left (**a**), the lesion is placed in the middle of the box, while on the right (**b**), the lesion is placed at the lower right border

The study was registered at clinical trials.gov (NCT02024113) and was approved by the Medical Ethics Review Committee of the Amsterdam UMC and registered in the Dutch trial register (trialregister.nl, NTR3508). All patients gave informed consent for study participation and use of their data for (retrospective) scientific research. Two datasets acquired at two institutions were included in this study with both datasets following the recommendations of the EARL accreditation program [19, 20]. All images were converted to Standardized Uptake Value (SUV) units before the segmentation process started in order to normalize the images for differences in injected tracer dose and patient weight. This paper focuses on the segmentation process and not on lesion detection. Therefore, before the start of the segmentation process, a large bounding box was drawn around every lesion including also a large number of non-tumor voxels as illustrated in Fig. 1. The bounding box was drawn randomly such that the tumor was not always appearing in the middle but on different locations in the box. This step was performed in order to avoid that the CNN remembers the location of the object instead of other, more important characteristics. As a CNN requires that all images have the same size, each bounding box had a size of 64 × 64 × 64.

#### Training and testing dataset

For training, validating and testing the segmentation approaches, 96 images of patients with NSCLC Stage III–IV were included. Patients fasted at least six hours before scan start and were scanned 60 min after tracer injection. All images were acquired on a Gemini TF Big Bore (Philips Healthcare, Cleveland, OH, USA). For attenuation correction, a low dose CT was performed. All images were reconstructed to a voxel size of 4 × 4 × 4 mm using the vendor provided BLOB-OS-TOF algorithm. More details about the patient cohort can be found in previous studies [21]. Fivefold cross-validation was performed whereby randomly 70% of the images were used for training, 10% for validation and 20% for independent testing.

#### Test–retest dataset

For a fully independent test–retest evaluation, ten PET/CT scans of patients with Stage III and IV NSCLC were analyzed. These ten patients underwent two whole-body PET/CT scans on two consecutive days. Images were acquired on a Gemini TF PET/CT scanner (Philips Healthcare, Cleveland, OH, USA) at a different institution (Amsterdam University Medical Center). Patient fasting time, time between tracer injection and scan start, as well as reconstruction algorithm and voxel size were the same as in the previous described dataset. A total of 28 lesions were included in the analysis.

#### Reference segmentations

The reference segmentations used for training, validating and testing the algorithm were obtained by applying an automatic segmentation which identified all voxels with a SUV above 2.5 as tumor (here after SUV2.5). The segmentations were manually adjusted by an expert medical physicist (RB) with more than twenty years of experience in PET tumor segmentation. This approach was chosen as it has been demonstrated that the manual adaption of a (semi-) automatic algorithm is more robust than a pure manual segmentation [22].

### Segmentation algorithm

All segmentation algorithms were implemented in Python 3.6 using the libraries keras and scikit-learn.

#### Convolutional neural network (CNN)

**Fig. 2:**
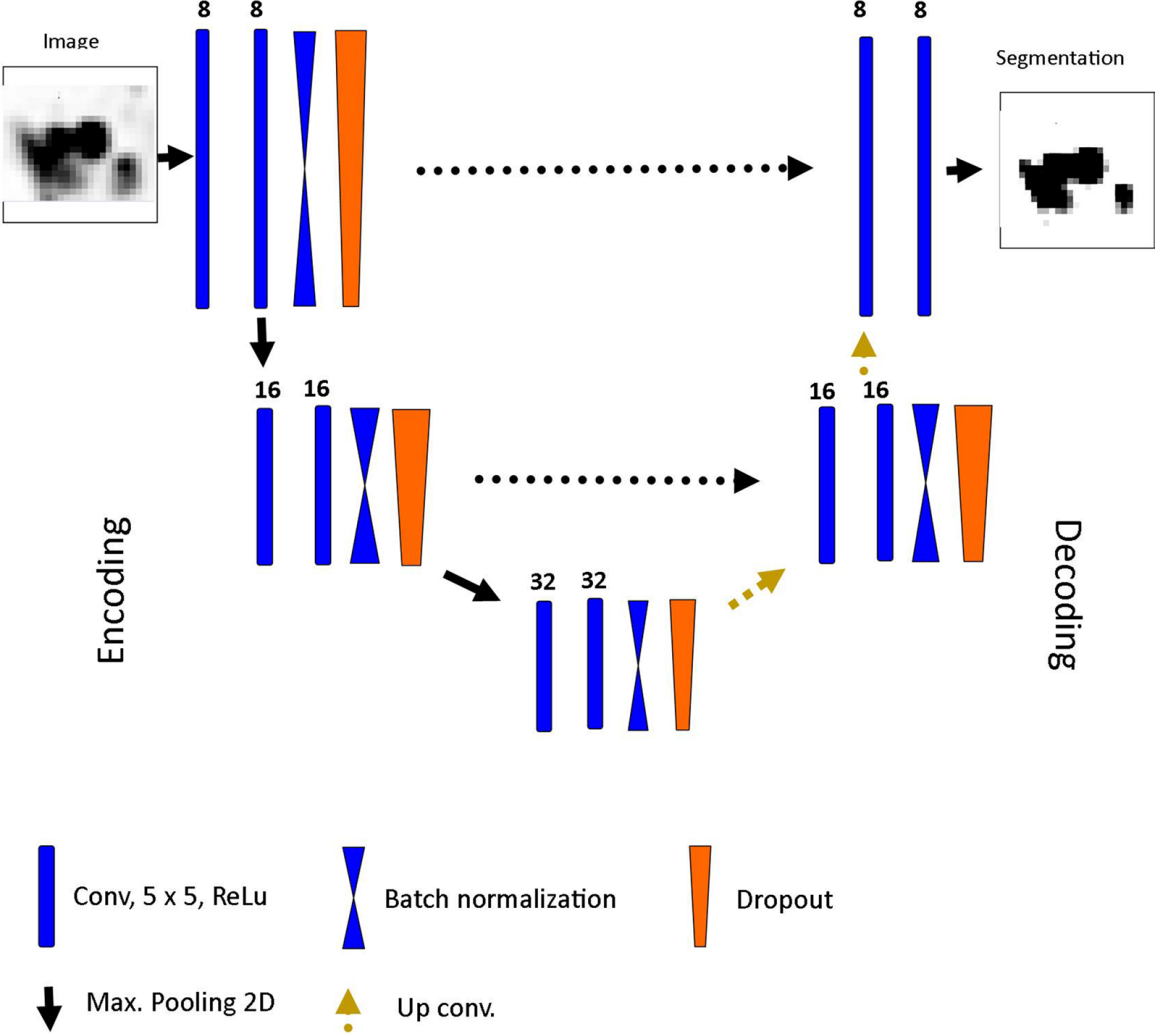
CNN architecture: In the encoding path, the images are subsequently downsampled while the number of features increases. In the decoding path, the images are upsampled while the number of features decreases. Encoding and decoding path are connected with skip connections

A 3D CNN following the U-Net architecture proposed by Ronneberger et al. [23] was implemented with the keras library. U-net is one of the most famous and most frequently used CNN architectures for biomedical image segmentation, and it was especially designed for scenarios where only a small number of training examples are available. An illustration of the used architecture is displayed in Fig. 2. An U-Net consists of an encoding and decoding part. In the encoding part, the images are subsequently down-sampled while the number of features is increased. In the decoding part, the images are up-sampled while the number of features decreases. In both parts, three layers consisting of one convolutional block (= two convolutional layers with a kernel size of 5 followed by a Rectified Linear Unit (ReLu) layer), a max-pooling layer for down-sampling in the encoding or a convolutional up-sampling layer in the decoding part, a batch normalization layer to increase network convergence and a drop-out layer to avoid overfitting. Due to the relatively small dataset, the CNN was trained with 8 initial features in the first layer. The number of layers and initial features were determined iteratively until the validation accuracy was optimal and at the same time comparable to the accuracy in the training set. The latter is important as a large difference in training and validation accuracy is a hint for overfitting. Details about training and validation accuracy for different number of initial features can be found in the Additional file 1: Tables S1 and S2. The CNN was trained for 1000 epochs with a batch size of 25. The learning rate was set to 0.001 and an Adam-optimizer was used for weight adaptation. The negative Dice-coefficient was used as loss function measuring the overlap of two segmentations. A Dice coefficient of 1 is reflecting a perfect overlap. A U-Net requires that all images have the same size.

In order to increase the amount of training data and to avoid over-fitting, data augmentation was performed. This included rotations within − 20° to 20°, shifting in width and height direction within 20% of the side length, a rescaling of the images within 25%, intensity stretching, as well as adding Gaussian noise to the image.

For training, testing and applying the CNN, the dataset was divided into smaller (≤ 12.8 ml) and bigger tumors. The threshold was chosen empirically, and it was found that this threshold led to the best performance. For each tumor size category, one separate CNN was trained. Splitting the dataset by lesion size was performed as this led to more accurate and repeatable segmentations (illustrated in Additional file 1: Section 4). In order to train the two separate networks, lesions were selected using the volume of the ground truth mask. Depending on this tumor size, the lesion was used for training the corresponding CNN. After training and testing the CNNs, the appropriate CNN for a specific lesion was selected based on an initial guess of the tumor size. The latter is obtained using a majority vote (MV) segmentation. This MV segmentation uses four standard threshold approaches as input (see explanation below and Additional file 1: Section 5). The MV segmentation was chosen for this task because it provided the most accurate segmentations when compared with manual segmentations in previous work [7], and it is easy to implement. This initial tumor MV segmentation was only performed to select the corresponding CNN, i.e., to distinguish between smaller and bigger lesions.

#### Textural feature segmentation (TF)

**Fig. 3:**
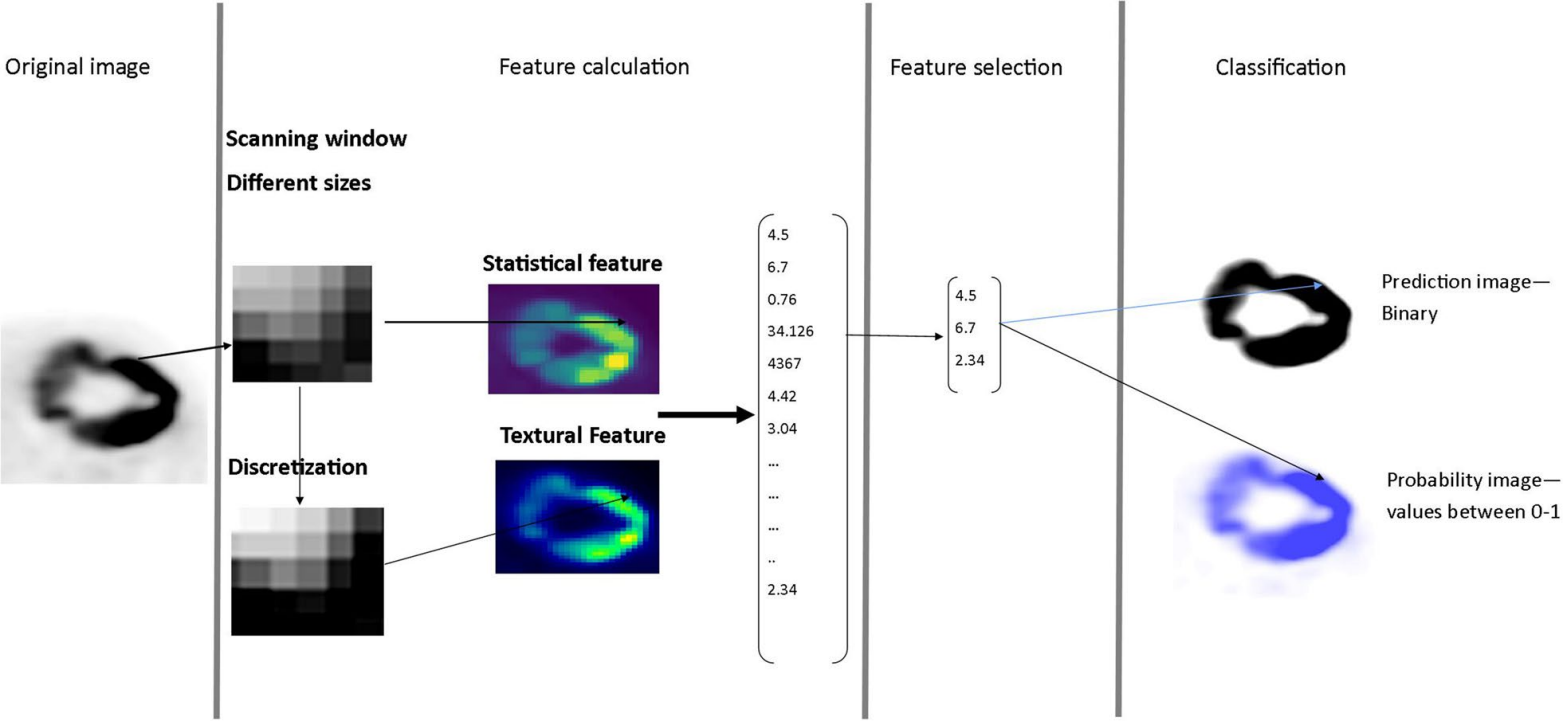
Workflow of the textural feature-based segmentation for the axial view

In the TF segmentation approach, textural features of voxel neighborhoods were used for the voxel-wise segmentation of the tumor. For every view (axial, sagittal, coronal), a separate segmentation was performed, and the summed probability was used to generate the final segmentation. The workflow of the TF segmentation for one image view is illustrated in Fig. 3. As illustrated, every voxel was regarded as center of a scanning window. For each scanning window, statistical and textural features were calculated using the open-source software pyradiomics [24]. The feature space was then reduced by selecting the most important features for the segmentation task, which were identified by a random forest.

Next, a random forest classifier was trained to classify each voxel as tumor or non-tumor. The trained random forest was then applied to the testing dataset. The probability images of the three orientations are summed in order to obtain the final classification. A probability image contains information on the certainty of the classifier making the right decision. All voxels with a summed probability of more than 1.8 were included in the final tumor segmentation. A more detailed description of the algorithm can be found in Additional file 1 and in Pfaehler et al. [25].

To evaluate how well the AI-based segmentations were matching the reference segmentation, the overlap between the AI-based segmentations and those of the reference segmentation were analyzed using Jaccard Coefficients, as explained later.

#### Conventional segmentation algorithm

The repeatability of the AI-based segmentations was compared with two established segmentation algorithm:

- 41%SUV_MAX_: all voxels with intensity values higher than 41% of the maximal SUV value (SUV_MAX_) are regarded as tumor
- SUV4: all voxels with a SUV higher than 4 are included in the segmentation

Moreover, two majority vote (MV) approaches combining four frequently used thresholding approaches were included in the comparison. Both MV approaches were previously found to be more repeatable than conventional approaches [7]. The underlying segmentation algorithms were the above described SUV4 and 41%SUVMAX method as well as a segmentation including all voxels with a SUV above 2.5 and a 50% of SUVmax threshold-based segmentation with background correction. The two MV segmentation methods include:

- MV2: the consensus of at least two of the 4 standard approaches
- MV3: the consensus of at least three of the 4 standard approaches

### Evaluation of segmentation algorithm

For the evaluation of the segmentation algorithms, several metrics will be reported. Data analysis was performed in Python 3.6.2 using the packages numpy and scipy.

#### Accordance of AI segmentation and reference segmentation

In order to determine the accordance of the AI and reference segmentations, the Jaccard Coefficient (JC) was calculated. The JC is defined as the ratio between the intersection and the union of two labels and gives an indication about the overlap of the two labels:

$${\text{JC}} = \frac{A \cap B}{{A \cup B}}$$

A JC of 1 indicates perfect overlap, while a JC of 0 indicates that there is no overlap at all.

Furthermore, as the JC does not contain information about volume differences, the ratio between the volume of AI and reference segmentations were calculated: $\frac{{{\text{MATV}}_{{{\text{SEGM}}}} }}{{{\text{MATV}}_{{{\text{REF}}}} }}$. A volume ratio above 1 indicates an over- and a volume ratio below 1 an under-estimation of the volume. A ratio of 1 represents perfect alignment. Finally, the distance of mass (barycenter distance) of the segmentations was calculated. Hereby, a barycenter distance close to 0 indicates perfect agreement.

#### Repeatability evaluation

The repeatability of the segmentation approaches was evaluated by comparing the differences of segmented volumes across days. For this purpose, the percentage test–retest difference (%TRT) was calculated:

$${\text{TRT}}\% = \frac{{\left| {{\text{vol}}_{{{\text{Day}}1}} - {\text{vol}}_{{{\text{Day}}2}} } \right|}}{{({\text{vol}}_{{{\text{Day}}1}} + {\text{vol}}_{{{\text{Day}}2}} )/2}}*100$$

The %TRT measures the proportional differences in segmented volume between the two consecutive scans. Moreover, the repeatability coefficient (RC) which is defined as 1.96 × standard deviation (TRT%) was calculated. Additionally, intraclass correlation coefficients (ICC) were calculated using a two-way mixed model with single measures checking for agreement. An ICC between 0.9 and 1 indicates excellent and an ICC between 0.75 and 0.9 indicates good repeatability [26]. If a lesion was completely missed by one segmentation approach, it was discarded from the analysis to analyze the same dataset for all segmentation approaches.

The accuracy metrics of the AI-based segmentations as well as the TRT% of all approaches were compared using the Friedman test. The Friedman test is a non-parametric test, which does not assume a normal distribution of the data or independency of observations. It compares the rank of each data point instead of only comparing mean or median values. This means that if a segmentation algorithm provides consistently more accurate results, it will be ranked higher even if its mean or median are lower. As the Friedman test only contains information to show a significant difference in the data, a Nemenyi test was performed in order to assess which methods resulted in significant differences. P-values below 0.01 were considered as statistically significant. A Benjamini–Hochberg correction was applied in order to correct for multiple comparisons.

## Results

### Accordance reference: AI-based segmentation

**Fig. 4:**
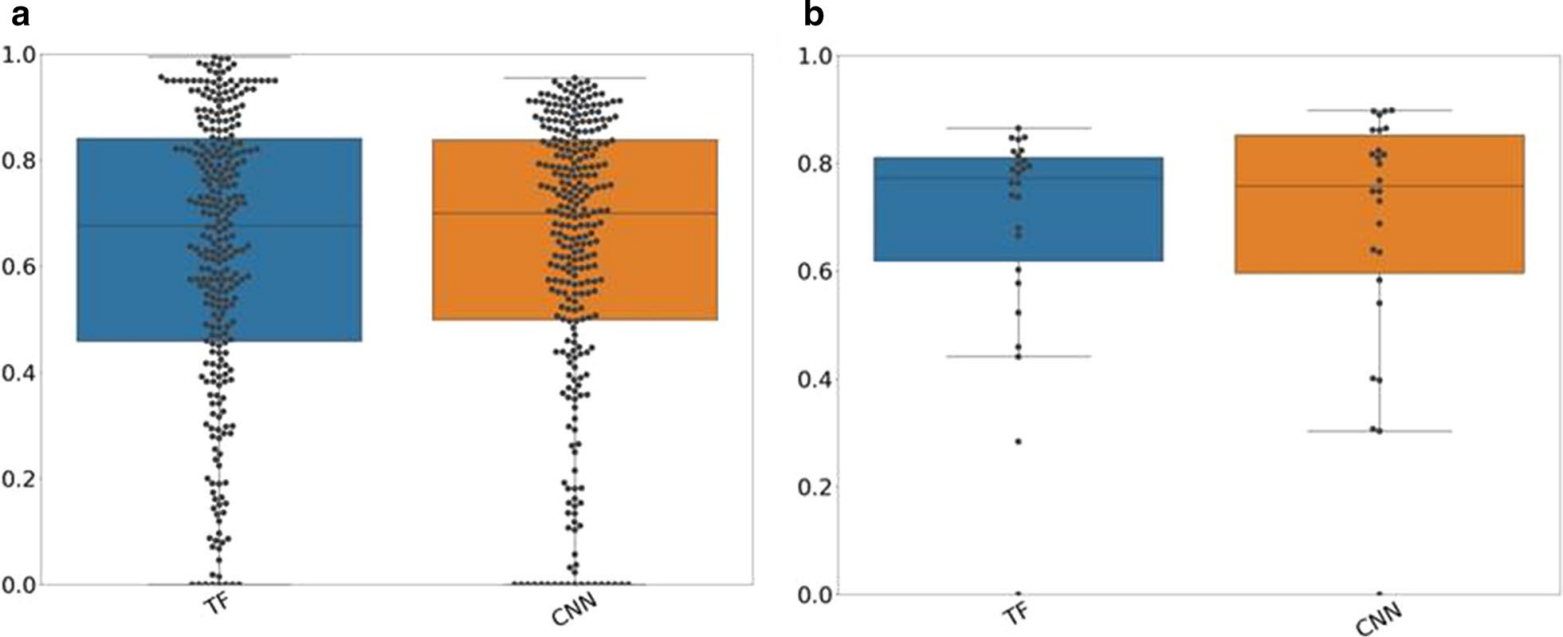
Jaccard coefficient (JC) values for both datasets: JC values for the cross-validated dataset (left figure) and the test–retest dataset (right figure) for the AI-based segmentation algorithm included in the study

Figure 4 displays the JC values between AI-based and reference segmentations for the cross-validation and test–retest dataset. The results of the separate folds are displayed in Additional file 1: Figure S8 and S9. In both cases, both approaches resulted in similar accuracies, which were not significantly different (*p* > 0.01). In the testing dataset, both approaches yielded good JC values (TF: median: 0.68, 25th percentile: 0.46, 75th percentile: 0.83, CNN: median: 0.7, 25th percentile: 0.52, 75th percentile: 0.84) indicating a good accordance with the reference segmentations. Volume ratios and barycenter distances are listed in Table 1. The CNN yields less underestimations and more overestimations of tumor volume [higher volume ratios (25th/75th percentile: 0.75/1.21)]. While the TF approach resulted in more underestimations of tumor volume (25th/75th percentile: 0.68/1.08). The barycentric distances of the TF approach were lower than the barycentric distances of the CNN. The corresponding values for the test–retest dataset can be found in Additional file 1: Table S3.

**Table 1:** Volume ratios and barycentric distances for TF and CNN

	Volume ratio Median (25th/75th quartile)	Barycentric distance Median (25th/75th quartile)
TF	0.70 (0.59/0.79)	0.68 (0.42, 1.66)
CNN	0.99 (0.83/1.34)	0.81 (0.36, 2.1)

**Fig. 5:**
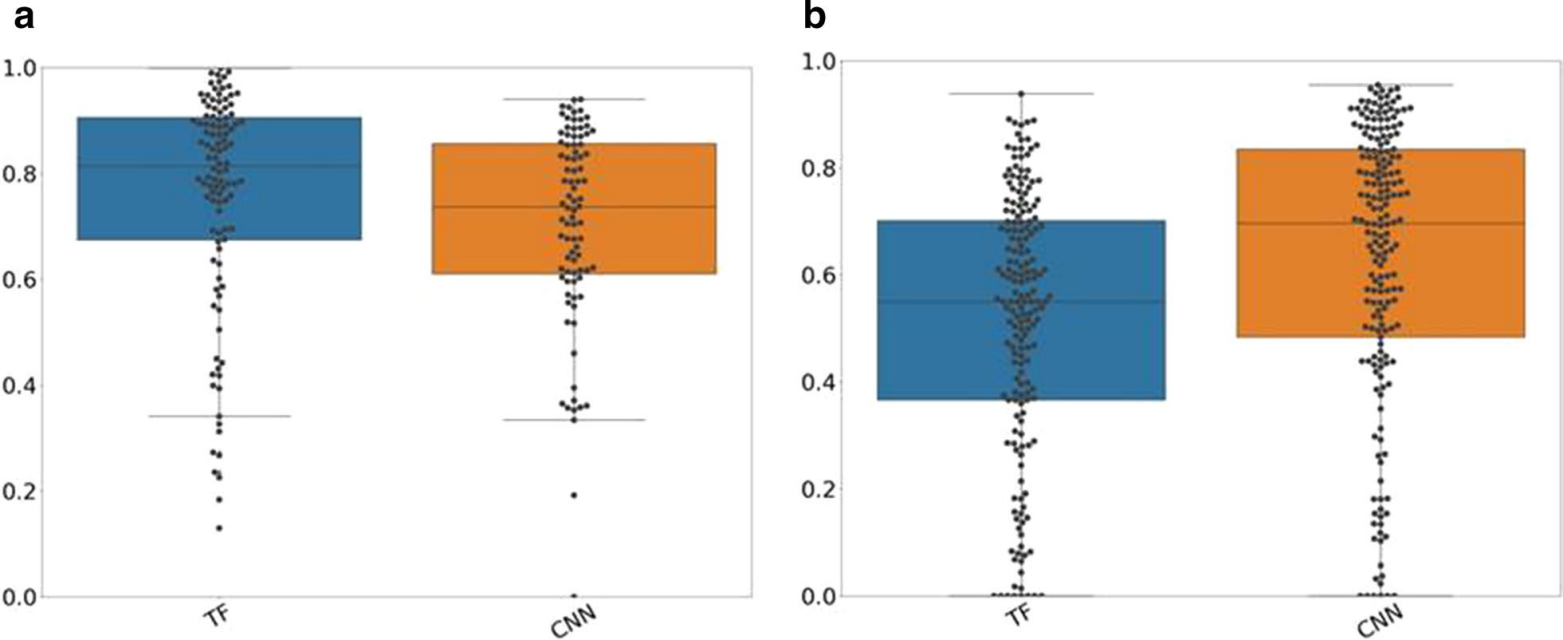
Jaccard coefficient (JC) values dependent on lesion size: JC values for bigger (left figure) and smaller (right figure) lesions for both AI-based segmentation approaches

In general, the accuracy of the segmentations depended on the lesion size as illustrated in Fig. 5. Segmentations of bigger tumors resulted in better accuracy than segmentations of smaller lesions. For larger lesions, the CNN resulted in a median JC value of 0.74, while the TF approach yielded a median JC of 0.82. For smaller lesions, the CNN yielded a median JC value of 0.7 which was higher than the median of the TF approach (0.56). For larger lesions, the CNN had a median volume ratio of 0.92 (25th/75th percentile: 0.81/1.13). While for smaller lesions, the CNN resulted in a median volume ratio of 0.92 (25th/75th percentile: 0.73/1.18). These results indicate that the CNN resulted in a similar number of overestimations for small and large lesions. While the TF approach yielded in the majority of the cases volume ratios below 0.8 and therefore for smaller and larger lesions more underestimations. All JC values, volume ratios and barycentric distances for smaller and larger lesions are listed in Table 2.

**Table 2:** Accuracy metrics for smaller and bigger lesions

	JC bigger Median (25th/75th quar)	Volume ratio diff bigger Median (25th/75th quar)	Barycentric distance Median (25th/75th quartile)	JC Median (25th/75th quar) smaller	Volume ratio smaller Med (25th/75th quar)	Barycentric distance Median (25th/75th quartile)
TF	0.82 (0.64/0.89)	0.91 (0.81/1.13)	0.61 (0.4/1.3)	0.56 (0.39/0.68)	0.91 (0.55/0.95)	0.76 (0.46/1.8)
CNN	0.74 (0.59/0.83)	0.92 (0.79/1.1)	1.1 (0.4/2.6)	0.7 (0.54/0.82)	0.92 (0.73/1.18)	0.77 (0.31/1.9)

As displayed in Fig. 5, TF and CNN resulted in three cases with JC values around or below 0.4 for bigger lesions. In these cases, the tumors were located close to the heart, which was incorrectly included in the segmentation. Therefore, the tumor volume was highly overestimated. A similar effect was observed for smaller lesions: The CNN did not segment some of the smaller lesions while this was not the case for the TF-based approach. All lesions that were completely missed were located close to the kidneys, which was wrongly identified as tumor. The TF approach segmented the kidney regions but still included the tumors in the final segmentation.

### Repeatability

**Fig. 6:**
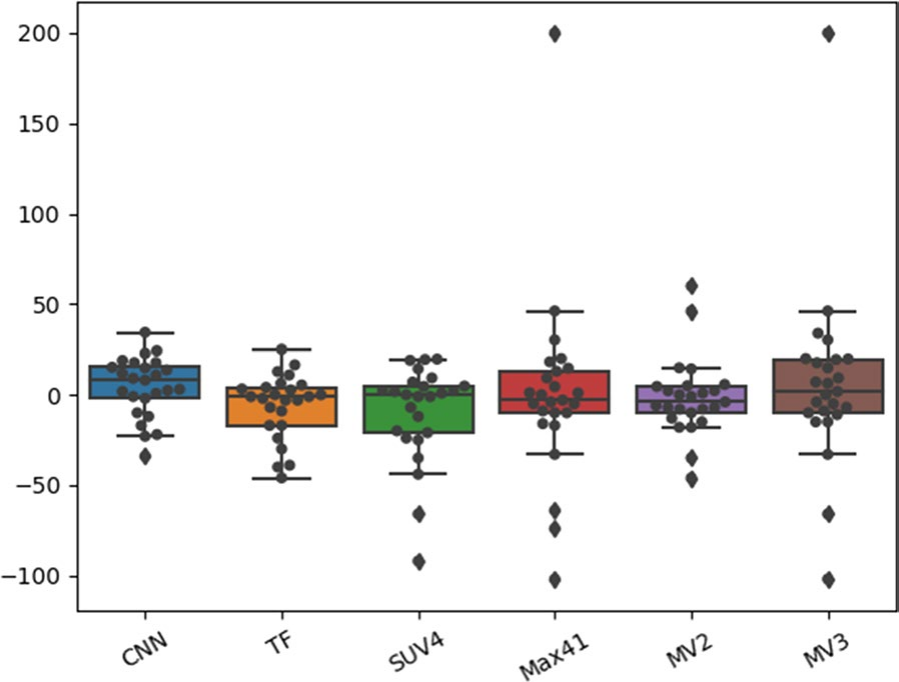
Test–retest coefficient (TRT%) for all segmentation approaches: If the TRT% is close to 0, the repeatability of the segmentations is excellent. Abbreviations of the segmentation algorithm: *SUV4* standardized uptake value 4, 41%SUV_MAX_, *MV2* majority vote 2, *MV3* majority vote 3, *TF* textural feature-based approach, *CNN* convolutional neural network

Figure 6 displays the TRT% for all segmentation algorithms. Two lesions were completely missed by the CNN and therefore discarded from the analysis.

CNN-based segmentations outperformed the other approaches regarding TRT% with an absolute mean value of 13.9% and a standard deviation of 16%. TF and MV2 segmentation yielded absolute mean values of 13.0% and 14.1% and standard deviations of 17% and 21%, MV3, 41%SUVMAX and SUV4 segmentations yielded mean values of 28.1%, 28.1% and 18.1%, and standard deviations of 50%, 51% and 26%. The corresponding repeatability coefficients can be found in Additional file 1: Table S4. After applying the Benjamini–Hochberg correction, the differences in TRT% were not significantly different.

The CNN resulted in a TRT% of more than 10% in 3 out of 28 cases, while the conventional methods resulted in a TRT% higher than 10% in 12 (MV2, SUV4, 41%SUV_MAX_) or 13 cases(MV3). The TF segmentation resulted in a TRT% of more than 10% in only 8 cases.

TF, CNN and MV2 yielded similar ICCs (TF: 0.98, MV2: 0.97, CNN: 0.99) indicating a very good repeatability. MV3, SUV4 and 41% SUVMAX resulted in ICC of 0.73, 0.81 and 0.68, respectively. The lesion size did not influence the repeatability of the segmentations.

### Summary of the results

In summary, CNN and TF segmentation resulted in a better repeatability when compared with conventional approaches. Furthermore, both approaches resulted in a good accuracy when compared with the reference segmentations. The observed differences between the two AI-based methods were not significant for accuracy nor for repeatability. Therefore, our results suggest that both AI methods are good candidates for the segmentation of NSCLC tumors in PET images and are more powerful than conventional approaches in terms of repeatability. However, use of these AI methods for other tumor types requires further validation and most likely additional (transfer or re-) training.

## Discussion

In this paper, we evaluated two AI-based segmentation approaches in terms of repeatability and analyzed their accordance with a reference segmentation. Both approaches resulted in a good accuracy when compared with the reference segmentation used. The differences in performance between both AI approaches were small and statistically non-significant.

The segmentation of smaller lesions remains also for these two AI approaches a challenging task. One reason might be that with decreasing tumor size, small misclassifications have a higher impact on accuracy metrics as illustrated in Additional file 1: Table S5. Smaller lesions also typically show a lower tumor-to-background ratio and are therefore more difficult to segment. This might be the reason that the CNN was not able to delineate some smaller lesions completely. Moreover, some metastasis are located close to other high-uptake regions (such as the kidney) and distinguishing tumor from normal uptake is in this case challenging for any segmentation algorithm and often requires manual correction. Especially for the CNN, the different locations of the metastasis and therefore the differences in surrounding tissue yield a more challenging learning task than the segmentation of primary lung tumors alone.

In terms of accuracy and precision, the CNN trained and tested in this study was comparable with previous CNNs designed for the segmentation of primary tumors in PET images. An important difference between our methods and other published algorithm is that our approaches rely on the PET image information only and can therefore also be used when only a low-dose CT is acquired aside of the PET image [14, 16]. Previous studies reported low segmentation performance when using the PET image for segmentation only [16, 18].

The CNN used in this paper is implemented with a relatively low number of features and layers when compared with the original U-Net or other CNNs designed for the segmentation of tumors in medical images [15, 23]. Due to the relatively small dataset in the present study, we found that these combination of numbers of features and layers prevents the network from over-fitting while still yielding good results. A possible reason why the network performs well with a small number of features might be that in this study, the CNN is trained on tumor segmentation in a predefined bounding box and does not need to detect the tumor.

When the tumor was located close to other high uptake regions such as the heart or the kidneys, both segmentation approaches delineated this normal tissue high-uptake region as tumor. The standard automatic segmentation methods included in this study are mainly intensity driven. and they are therefore also not able to distinguish tumor from high-uptake regions when both are in close proximity. For these cases, it is likely that human interaction will always remain necessary, as mentioned previously [27]. However, in future studies, we will investigate if these segmentation approaches might also be used for lesion detection.

Also when compared with previous studies, the CNN and TF approaches outperformed other (semi-) automatic segmentation methods. Frings et al. reported a TRT% repeatability coefficient of 44.4–71.1 for all lesions included in their analysis when using different threshold-based segmentation approaches with background correction [28]. The AI-based segmentation methods yielded repeatability coefficients of 31.36 (CNN) and 33.36 (TF), which are better than those reported by Frings et al. For images acquired under the same conditions as in our study (i.e., 60 min time between tracer injection and scan start and EARL-compliant reconstructions), Kolinger et al. found repeatability coefficients of 43 to 56 *, which are also higher than the ones of our AI-based segmentations [7]. However, Kolinger et al. reported lower repeatability coefficients for MV3 and 41% SUV_MAX_ segmentation approaches. The reason for this might be that Kolinger et al. compared the repeatability for the summed MATV of all lesions (TMATV), while we compared the repeatability of MATV for each lesion separately. A discrepancy in the segmentation of one lesion, especially if the lesion is small, has less impact on the repeatability of TMATV.

A disadvantage of AI-based segmentation approaches is the need for reliable training data. The lack of reasonable training data is one drawback making the clinical implementation of AI-based segmentation algorithms challenging. However, the MV2 approach used in this study was found to result in accurate and robust segmentations in a previous study [7]. Moreover, in our study, it also outperformed the conventional segmentation approaches in terms of repeatability without depending on training data. Especially for tasks where segmentation accuracy is important, such as radiotherapy planning, the MV2 is a good candidate for clinical use. Yet, regardless the method used, the final segmentation should always be supervised. In terms of repeatability, the CNN segmentation outperformed the MV2 approach and is the method of choice when segmentation repeatability is important, such as for longitudinal studies and/or for the evaluation of treatment response.

Another drawback of AI-based segmentation approaches is that they are trained for one specific task such as the segmentation of lung tumors or head and neck tumors. To apply the already trained algorithms to other (even similar tasks) requires a re-training of the algorithms. Therefore, both segmentation algorithms trained and validated in this study are likely not directly applicable to other cancer types such as head and neck cancer. Before using them for another cancer types, the algorithms need to be re-trained or at least undergo rigorous validation.

One limitation of this study is that the reference segmentations were delineated by one, yet experienced, observer while the consensus of three expert segmentations is considered as gold standard. To account for this, the segmentation was initiated with a semi-automated delineation method, an approach known to reduce observer variability. Of note, for the test–retest study, the same lesions were delineated by 5 observers in a previous study (7), and it was shown that even the consensus contour of these observers was less repeatable than those seen with any of the automated approaches. Finally, in our repeatability study, we included the AI-based approaches as well as several conventional methods and this repeatability study showed that our trained AI approaches provided very good results, even if the ground truth segmentations used during training of the AI methods would have been suboptimal.

Another limitation is the small dataset used for repeatability analysis. However, the collection of test–retest scans is unfortunately limited due to the patient burden coming with consecutive scans of the same patients. Future studies, especially studies using data from different centers should confirm our findings.

## Conclusion

In this paper, we compared the repeatability of AI-based segmentation algorithm with conventional segmentation approaches. Our results illustrate the advantage of AI-based segmentation approaches: Both approaches resulted in a good accuracy when compared with the reference segmentation and showed a high repeatability. Together with a majority vote approach (combining the results of four conventional segmentation approaches) the proposed AI-based segmentation methods were superior to the other segmentation algorithms included in this study in terms of repeatability. This study demonstrates that AI-based segmentations have not only the potential to accurately segment lesions but also result in more repeatable segmentations.

## Supplementary Information

**Additional file 1:** Supplemental material.

## Data Availability

The datasets used and/or analyzed during the current study are available from the corresponding author on reasonable request.

## Abbreviations

PET: Positron emission tomography
MATV: Metabolic active tumor volume
TMATV: Total MATV
TLG: Total lesion glycolysis
TF: Textural feature
CNN: Convolutional neural network
JC: Jaccard coefficient
TRT: Test–retest coefficient
ICC: Intraclass correlation coefficient
FDG: Fluorodeoxyglucose
CT: Computed tomography
AI: Artificial intelligence
NSCLC: Non-small cell lung cancer
SUV: Standardized uptake value
MV: Majority vote
