# Supplementary material for: Repeatability of two semi-automatic artificial intelligence approaches for tumor segmentation in PET
Source: EJNMMI Res. 2021 Jan 6;11:4. doi: 10.1186/s13550-020-00744-9 (PMC7788118; doi:10.1186/s13550-020-00744-9)
Supplement: Supplementary file 1 — Additional file 1: Supplemental material. [file 13550_2020_744_MOESM1_ESM.docx]

**Supplemental Material**

1. **CNN Training Details**

Number of layers, optimal kernel size, initial learning rate, and dropout rate were experimentally determined using the training set. For this purpose, an iteration was performed using different values for these variables. The variables leading to the best performance in the training set were used.

Number of layers, optimal kernel size, initial learning rate, and dropout rate were experimentally determined using the training set. For this purpose, an iteration was performed using different values for these variables. The variables leading to the best performance in the training set were used.

Hereby, the number of initial features, as well as the number of layers was iteratively changed. The number of features and layers leading to no overfitting and at the same time to the highest Dice coefficient in the validation set were used. Table S1 and S2 list the training and validation accuracy for the different number of initial features and layers.

| **Nr of layers** | **Nr of initial features** | **Training accuracy** | **Validation accuracy** |
| --- | --- | --- | --- |
| 3 | 8 | 81 | 80.5 |
| 3 | 16 | 81.4 | 79.4 |
| 3 | 32 | 83.2 | 77.3 |
| 3 | 64 | 85.1 | 75.6 |
| 3 | 128 | 87.1 | 71.1 |
| 3 | 256 | 89.1 | 69.4 |

Table S1: Training and validation accuracy when varying the inital number of features

| **Nr of layers** | **Nr of initial features** | **Training accuracy** | **Validation accuracy** |
| --- | --- | --- | --- |
| 3 | 8 | 81 | 80.5 |
| 4 | 8 | 75.4 | 71.4 |
| 5 | 8 | 65.2 | 62.3 |
| 3 | 16 | 81.4 | 79.4 |
| 4 | 16 | 74.3 | 70.1 |
| 5 | 16 | 58.2 | 59.1 |

Table S2: Training and validation accuracy when varying the number of layers for 8 and 16 initial features

# **Details TF**

#
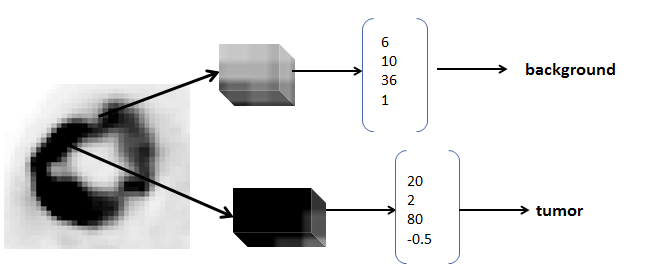


Figure S2: Illustration of textural feature based segmentation: For each voxel a scanning window is defined. From this scanning window, features are calculated and then used for classification.

# The general idea behind the textural feature based approach is illustrated in Figure S2. For every voxel a neighborhood is defined. From this neighborhood features describing the intensity distribution and the texture are calculated. These features are then used for classifying each voxel as tumor or background.

#
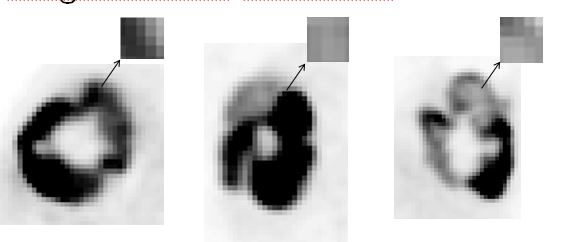


Figure S1: Illustration of the definition of 2D neighborhoods for each view separately. As illustrated, each view contains different information.

# In our approach, we take advantage of the fact that the different views (axial, coronal, sagittal) contain different information as illustrated in Figure S3. For each view, a separate neighborhood is defined and features are calculated. In a second step, feature selection is performed for each view separately using a random forest. The random forest gives information on how important a feature is for the classification task. The six most important features were kept and used for training and applying the classifier. Feature selection was performed for every view separately, but the selected features were consistent for all views. The features max, energy were selected.

# For every voxel, a feature vector containing the six most important features, was used for training, validating and testing the classifier.

#
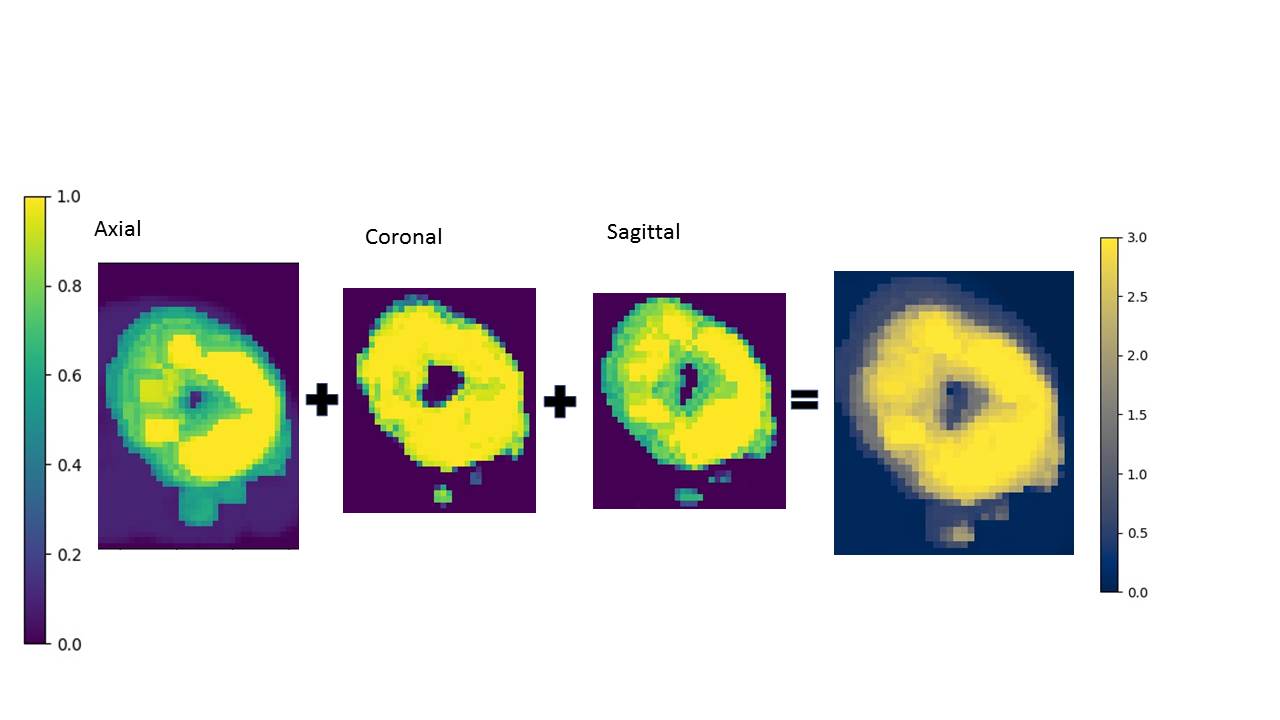


Figure S2: Combination of probability images leads to final segmentation

# After the classifier was applied, the probability images of the random forest were combined as illustrated in Figure S4. A voxel yielding a summed probability above 1.8 was regarded as tumor and included in the segmentation

# **Cross-validation results**

# **
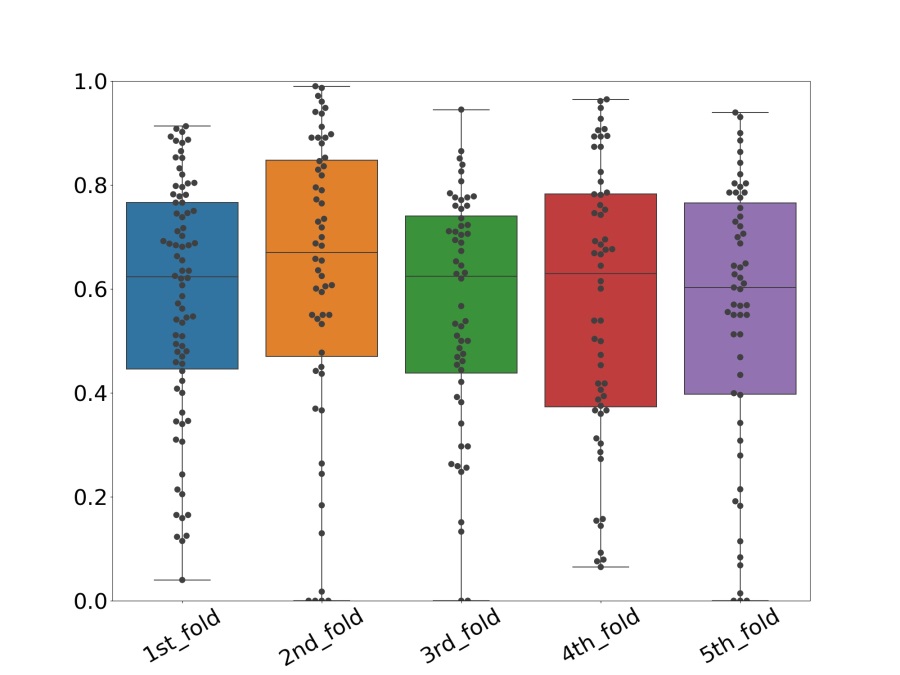
**

# **
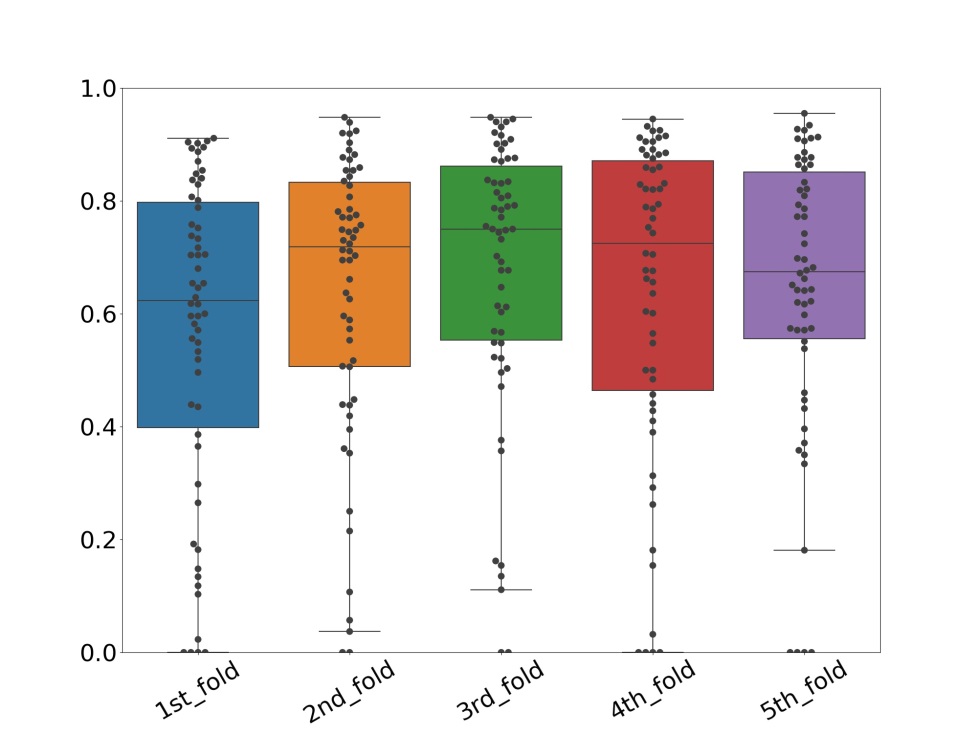
**

# **Analysis of algorithm performance - CNN**

# This section contains a description of the performed experiments with the two AI based segmentation methods in order to justify the choices for the construction of the TF algorithm and the training sets of the CNN. The best performance of the CNN was achieved when training two separate networks: one for smaller and one for bigger tumors. However, also other combinations of datasets were considered for training the CNN:

# Train network only on bigger lesions

# Train network only on smaller lesions

# Train network on all training data (independent of lesion size)

# Train network on a balanced training data set (equal number of smaller and bigger lesions)

-
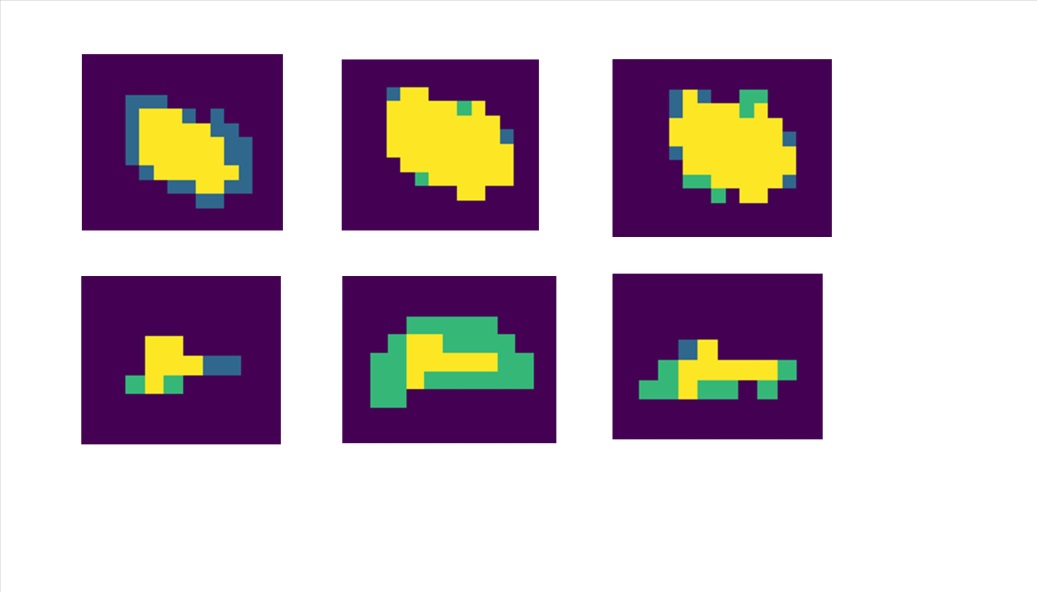

- Figure S5: Illustration the impact of the different training scenarios on the segmentation results: Left: network trained only on smaller tumors, Middle: Results when trained only on bigger tumors, Right: Trained on all tumors; Yellow: true positives, Purple: true negative, Blue: False negatives, Green: False positives

# As displayed in Figure S**5**, the networks trained on a special lesion size yielded only good results for the same lesion size, while it was failing for the other lesions. When trained on bigger lesions, smaller lesions were either missed or overestimated. While when trained on smaller lesions, only the very high uptake parts of the bigger lesions were segmented.

# The networks trained on the pulled data did not lead to satisfactory results. When trained on all data, the bigger lesions were over-segmented while several smaller lesions were not detected. Also training the CNN on a balanced dataset did not improve segmentation accuracy.


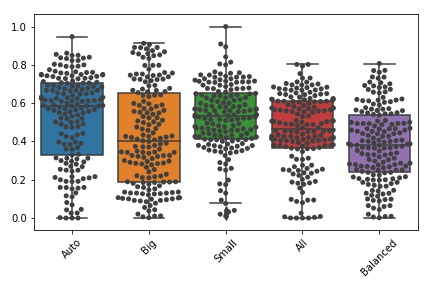


Figure S6: Jaccard Coefficient (JC) for different CNN training sets: Auto: Automatic selection of the right network as proposed in this paper; Big: Trained only on bigger lesions, Small: Trained only on smaller lesions, All: Trained on all lesions, Balanced: Trained on a balanced dataset with an equal number of small and big lesions


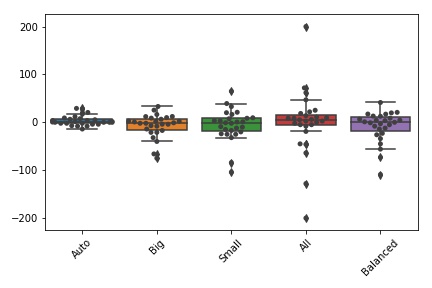


Figure S7: Test-retest coefficient (TRT%) for different CNN training sets: Auto: Automatic selection of the right network as proposed in this paper; Big: Trained only on bigger lesions, Small: Trained only on smaller lesions, All: Trained on all lesions, Balanced: Trained on a balanced dataset with an equal number of small and big lesions

# The overall best accuracy and repeatability was achieved when using two separate CNNs and selecting the appropriate CNN for the actual lesion automatically as illustrated in Figure S**6** and S**7**.

1. **Threshold-based approaches included in Majority Vote**

Four threshold based approaches were included in the majority vote segmentation:

- SUV2.5: All intensity values with a SUV above 2.5 are regarded as tumor
- SUV4: All intensity values with a SUV above 4 are regarded as tumor
- 41%SUVMAX: The highest intensity value in the pre-defined mask is determined (SUVMAX). All voxels with intensity values above 41% of the SUVMAX are regarded as tumor.
- AUTO: The SUV_PEAK_ is calculated as well as the average background (bg) intensity. All intensity values above 50% of the (SUVPEAK – average(bg)) are regarded as tumor

1. **Results of individual folds**

The JC values of the different cross-validation folds are displayed in Figure S8 for the CNN and Figure S9 for the textural feature based approach..


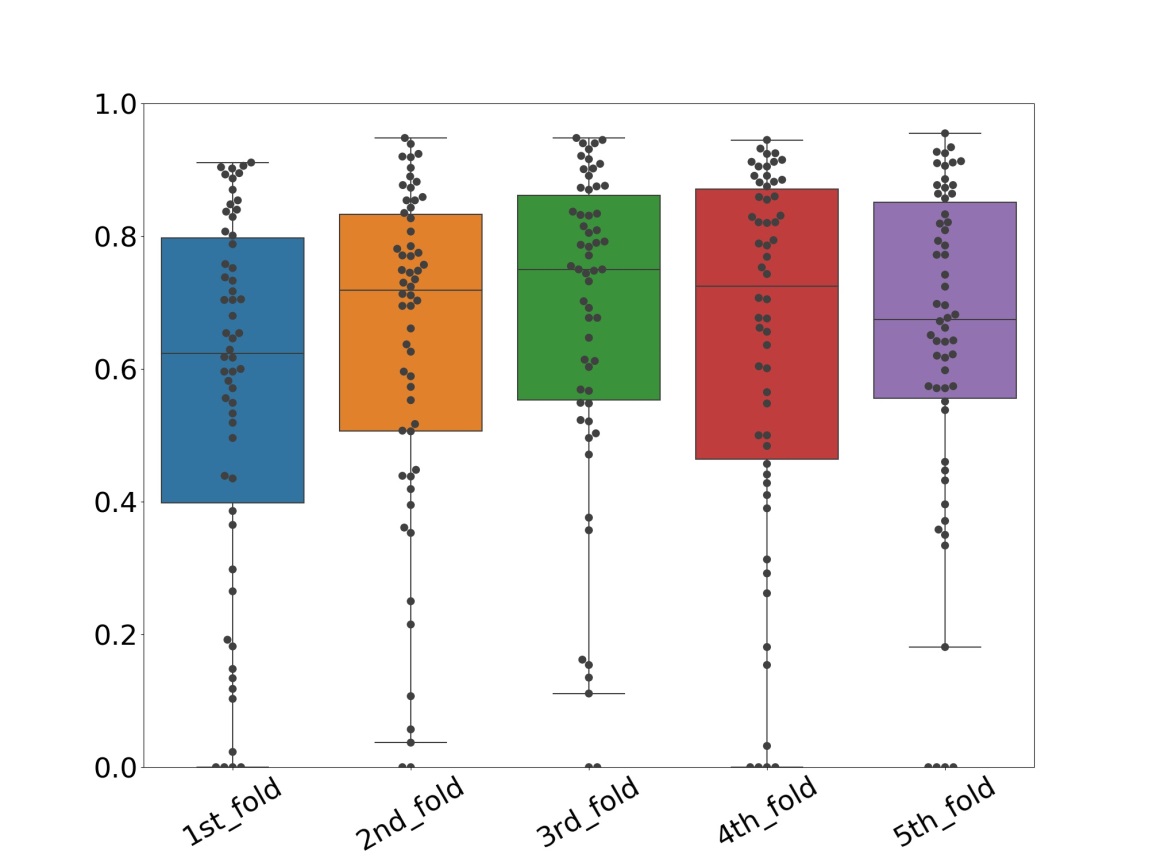


Figure S8: JC values of the separate cross-validation folds of the CNN


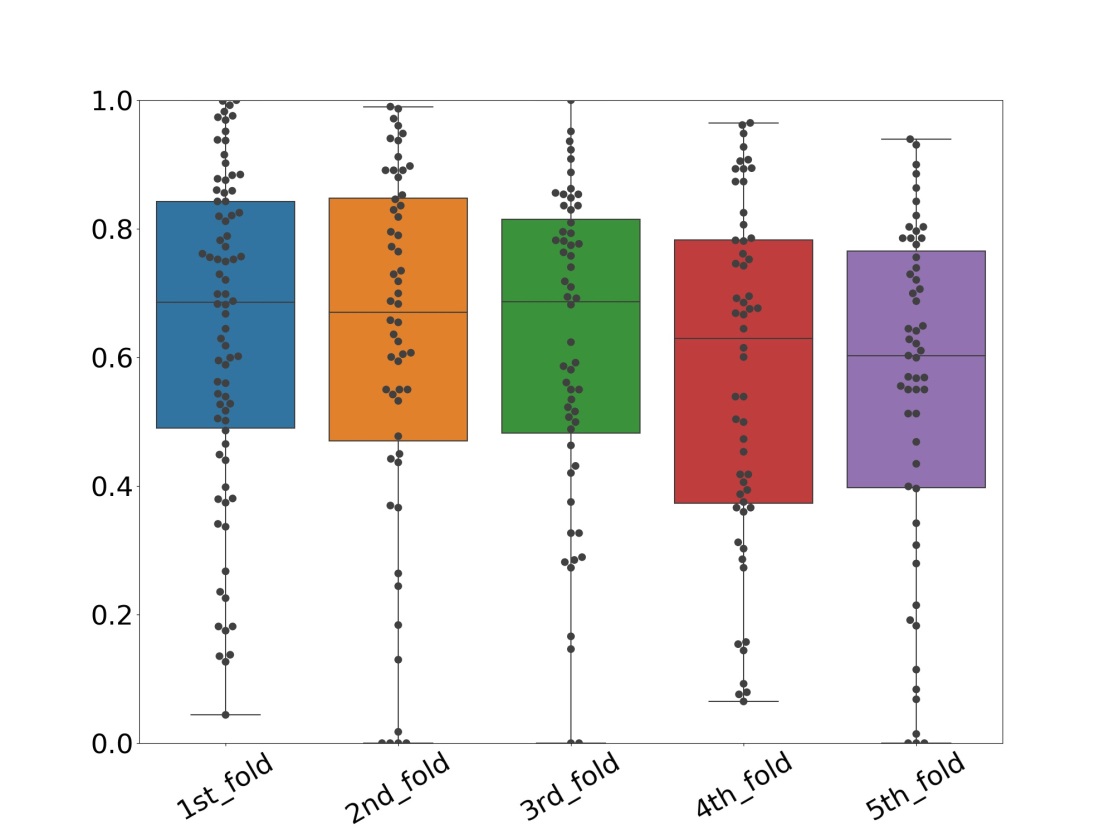


Figure 3: JC values of the separate cross-validation folds of the TF based segmentation approach

1. **Accuracy metrics for test-retest dataset**

|  |  | Volume ratio  median (25^th^/75 ^th^ percentile) | Barycentric distance  median (25 ^th^ /75 ^th^ percentile) |
| --- | --- | --- | --- |
| TF | 0.77 (0.62/0.81) | 0.92 (0.77/1.01) | 0.59 (0.49/2.14) |
| CNN | 0.76 (0.60/0.85) | 0.90 (0.80/0.99) | 0.54 (0.25/3.45) |

Table S3: Accuracy metrics for TRT-dataset

1. TRT% values

|  | Mean (Median) TRT% | Std TRT% | 25^th^/75^th^ percentile TRT% |
| --- | --- | --- | --- |
| SUV4 | 18.1 (5) | 26 (50.96) | -21.1/4.8 |
| MV3 | 28.1 (20) | 50 (98) | -10.0/19.5 |
| MV2 | 14.1 (5) | 21 (41.16) | -10.0/4.6 |
| 41%SUV_MAX_ | 28.4 (15) | 51 (99.94) | -10.0/13 |
| TF | 13.0 (-1) | 17 (33.32) | -17.1/4.1 |
| CNN | 13.9 (8) | 16 (31.36) | -2.1/15.2 |

Table S4: Mean and standard deviation of TRT

1. **JC values and volume ratio dependent on tumor size**

The table displayed below illustrates the dependence of JC values and percentage volume differences on the tumor size. We simulated an experiment where the segmentation overestimated the tumor for exactly one voxel-layer. For this segmentation and the known ground-truth JC values and percentage volume differences are calculated. This experiment illustrates that for smaller lesions, this small segmentation error has a high impact on JC values and percentage volume differences, while for bigger lesions, the JC values are above 0.8.

| Tumor volume (ml) | JC | volRatio |
| --- | --- | --- |
| 3.84 | 0.23 | 4.42 |
| 9.024 | 0.32 | 3.12 |
| 16.96 | 0.39 | 2.54 |
| 28.16 | 0.45 | 2.26 |
| 43.136 | 0.50 | 2.00 |
| 62.4 | 0.54 | 1.86 |
| 86.464 | 0.57 | 1.75 |
| 115.84 | 0.60 | 1.66 |
| 151.04 | 0.63 | 1.60 |
| 192.576 | 0.65 | 1.54 |
| 240.96 | 0.67 | 1.50 |
| 258.304 | 0.69 | 1.46 |
| 360.32 | 0.70 | 1.42 |
| 432.32 | 0.72 | 1.40 |
| 513.216 | 0.85 | 1.18 |

Table S5: Jaccard coefficient and volume ratios in dependence of the original tumor volume
